# Efficacy and safety of parecoxib sodium for acute postoperative pain: A meta-analysis

**DOI:** 10.3892/etm.2013.1172

**Published:** 2013-06-20

**Authors:** WEI WEI, TIANYUN ZHAO, YUANTAO LI

**Affiliations:** 1 Department of Anesthesiology, Meizhou People’s Hospital, Meizhou, Guangdong 514031;; 2 Department of Anesthesiology, Shenzhen Maternity and Child Healthcare Hospital, Southern Medical University, Shenzhen, Guangdong 518028, P.R. China

**Keywords:** parecoxib sodium, postoperative pain, meta-analysis

## Abstract

This meta-analysis was performed to evaluate the efficacy and safety of parecoxib sodium for acute postoperative pain. PubMed, Cochrane Central Register of Controlled Trials, EBSCO, Springer, Ovid and Chinese National Knowledge Infrastructure (CNKI) databases were searched from January 1999 to January 2013 to comprehensively collect randomized controlled trials (RCTs) of parecoxib sodium for acute postoperative pain. The methodological quality of the included RCTs were assessed and the data were extracted by two reviewers independently according to the Cochrane Handbook. Efficacies and safety (respiratory depression, pruritus, fever, headache, and nausea and vomiting) were pooled using meta-analysis performed by Review Manager 5.1 software. Relative risk (RR) and 95% confidence interval (CI) were calculated in a fixed-effects model. Seven RCTs involving 1,939 patients met the inclusion criteria. The results of the meta-analysis revealed that the rate of ‘effective’ treatment as described by the patients’ global evaluation of study medication (PGESM) was higher in the patient-controlled analgesia (PCA) combined with parecoxib sodium group 24, 48, and 72 h after the initial intravenous dose of 40 mg parecoxib compared with that in the control group [PCA alone; RR=1.41, 95% CI (1.13–1.75); RR=1.25, 95% CI (1.15–1.35); and RR=1.30, 95% CI (1.21–1.40), respectively]. The rate of ‘ineffective’ treatment in the PCA combined with parecoxib sodium group was lower compared with that of the control group [RR=0.43, 95% CI (0.26–0.72); RR= 0.44, 95% CI (0.34–0.57); and RR= 0.33, 95% CI (0.23–0.48), respectively]. Combination of PCA with parecoxib sodium reduced the incidence of postoperative fever [RR=0.34, 95% CI (0.22–0.53)], as well as nausea and vomiting [RR=0.69, 95% CI (0.57–0.83)]; however, it did not significantly reduce respiratory depression [RR= 0.84, 95% CI (0.38–1.83)], pruritus [RR= 0.91, 95% CI (0.54–1.52)] or headache [RR=0.77, 95% CI (0.47–1.28)]. The combination of PCA with parecoxib sodium successively injected for <3 days significantly increases the scores of PGESM and reduces the incidence of adverse effects and postoperative complications.

## Introduction

Acute pain occurs as a result of tissue damage, often accidentally due to an injury or surgery. Acute postoperative pain is a manifestation of inflammation due to tissue injury. The management of postoperative pain and inflammation is a critical component of patient care (1).

Non-steroidal anti-inflammatory drugs (NSAIDs) are commonly used in the management of post-operative pain. NSAIDs inhibit cyclooxygenase (COX) enzymes, which are involved in the synthesis of prostaglandins and thereby reduce pain and inflammation. The inhibition of COX is the principal mechanism for the efficacy and the toxicity of NSAIDs (2) and it has been demonstrated that COX exists as at least two isoenzymes, COX-1 and COX-2 (3). Traditional NSAIDs non-specifically inhibit COX-1 and COX-2, whereas specific COX-2 inhibitors only affect the activity of COX-2. The major reason for development of specific COX-2 inhibitors was the maintenance of the anti-inflammatory and analgesic effects without altering the homeostatic functions of COX-1 (4). To represent an attractive alternative for patients requiring NSAIDs perioperatively, the selective COX-2 inhibitors, besides their improved side-effect profile, should have an equipotent analgesic efficacy relative to traditional NSAIDs.

Furthermore, oral NSAIDs are used post-operatively; however, when patients are unable to tolerate oral medications or require a faster onset of analgesia, parenteral administration may be preferred. Parecoxib is a COX-2 selective inhibitor, which may be administered as an intravenous or intramuscular injection for the short-term management of postoperative pain. It is a prodrug (the parent drug is inactive) that is rapidly hydrolysed *in vivo* to its active form, valdecoxib (5). Clinical trials have indicated that parecoxib is effective in treating postoperative pain resulting from oral surgery, orthopedic surgery and abdominal hysterectomy pain. Other studies have demonstrated no significant effects on platelet function or upper gastrointestinal mucosa (6–9). As a result, parecoxib sodium has been approved in European countries for the treatment of postoperative pain.

The combination of PCA and the selective COX-2 inhibitor parecoxib has reportedly been used for acute postoperative pain for years in European countries; however, the efficacy and safety of the combination has not yet been investigated. Therefore, to investigate the efficacy and safety profile of the combination of PCA and parecoxib for postoperative analgesic effects, we conducted a meta-analysis of randomized controlled trials (RCTs).

## Materials and methods

### Search sources and strategy

The search strategy was produced according to working handbook version 4.2.7 from the Cochrane collaboration (10). Studies were identified by extensively searching PubMed, Cochrane Central Register of Controlled Trials, EBSCO, Springer, Ovid and Chinese National Knowledge Infrastructure (CNKI) databases from January 1999 to January 2013. In addition, a manual search of abstracts from selected conferences was conducted, as well as a search by hand of the bibliographies of all relevant trials. The following search criteria were used: ‘parecoxib sodium’, ‘cyclooxygenase-2 inhibitor’ and ‘RCTs’. The language of the studies was not restricted to English.

### Study selection

Two reviewers independently conducted the literature search and extraction of relevant articles. The title and abstract of potentially relevant studies were screened for appropriateness before retrieval of the full articles. Any disagreement concerning study selection or data extraction was resolved by consensus with the third reviewer. For meta-analysis, all studies had to meet the following inclusion criteria: i) a study described as an RCT; ii) patients with no statistically significant differences in baseline characteristics; iii) intervention: a) treatment group, PCA combined with parecoxib sodium (successively injected for <3 days) intravenously at 40+20/40 mg bid; b) control group, same volume of saline; and iv) outcome variables: a) according to patients’ global evaluation of study medication (PGESM), pain relief 24, 48 and 72 h after the initial intravenous dose of 40 mg parecoxib was assessed on a four-point scale (0, none; 1, a little or some; 2, a lot; and 3, complete; scores 1 and 2 were defined as ‘ineffective’ and 3 and 4 were defined as ‘effective’); b) adverse reactions of opioids, including respiratory depression, pruritus, fever, headache, nausea and vomiting.

The exclusion criteria were as follows: i) a single injection of parecoxib sodium before PCA; and ii) PCA not combined with parecoxib sodium following surgery.

### Data extraction and assessment of study quality

Two of the authors independently extracted data from the trials that met the inclusion criteria. Authors were contacted for missing data when necessary. From each study, the following information was extracted: author, year of publication, sample size and intervention measures.

Quality assessment of the RCTs included in the meta-analysis was independently performed by the same reviewers according to the Cochrane Handbook 5.0.1 and Juni *et al* (11,12). Jadad grade was evaluated using the following items: i) was the study a randomized trial; ii) was the randomization scheme described and appropriate; iii) was the study described as double-blinded; iv) was the method of double-blinding appropriate; v) was there a description of allocation concealment; vi) was there a description of dropouts and withdrawals; and (vii) did the patients have statistically significant differences in baseline characteristics. Each author rated the quality of the trials using Jadad grade (maximum grade, A; minimum grade, C; grade ≥B, good quality).

### Statistical analysis

Data were analyzed using Review Manager 5.1 (provided by The Nordic Cochrane Centre, The Cochrane Collaboration). Included articles were pooled and weighted (13). Relative risk (RR) and 95% confidence interval (CI) were calculated in a random-effects model or in a fixed-effects model. Heterogeneity was assessed by χ^2^ test and the quantity of heterogeneity was measured with I^2^ statistic. If heterogeneity (P<0.01 or I^2^>50%) was identified among the trials, a random-effects model was selected, otherwise a fixed-effects model was selected. If heterogeneity was evident (I^2^>70%), the inferior quality study was eliminated for analysis.

## Results

### Study characteristics

**Figure 1.**
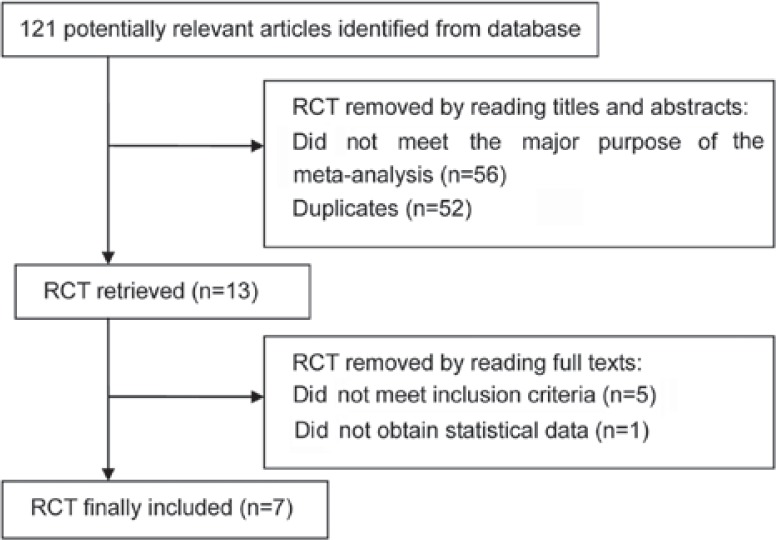
Study selection process. RCT, randomized controlled trial.

There were 121 articles relevant to the search terms and seven articles (14–20) involving 1,939 patients (treatment group, 1,207 patients; control group, 732 patients) were included in this meta-analysis. The flow chart for the selection of RCTs is shown in Fig. 1. The characteristics of the included trials are shown in Table I.

### Methodological quality assessment

The quality assessment of included RCTs is presented in Table I. All the trials included in this meta-analysis clarified adequate randomization procedures, used double-blinding and reported numbers of dropouts/withdrawals during the treatment; however, no study reported allocation concealment clearly. According to the Jadad score, all studies were eventually assessed to be good in terms of methodology with Jadad score B.

### Comparisons of effectiveness

#### Patient global evaluation 24 h after the initial dose of parecoxib

**Figure 2.**
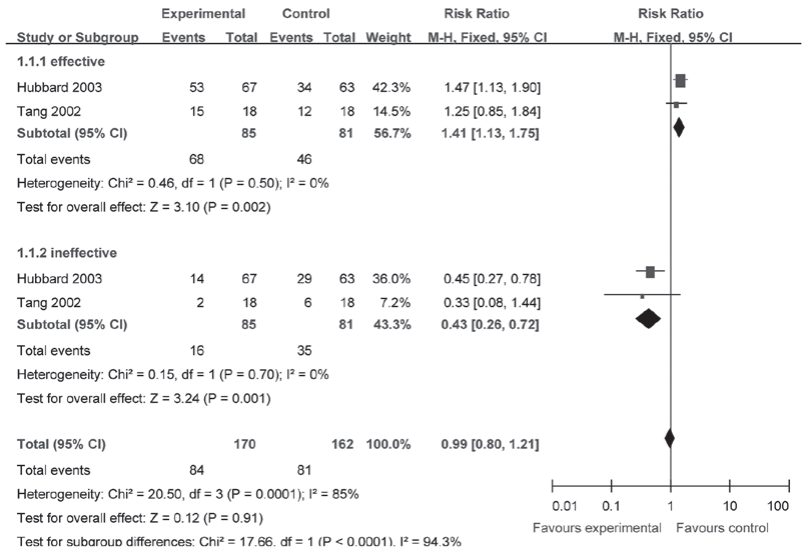
Meta-analysis of patients’ global evaluation of study medication of PCA combined with parecoxib sodium and PCA alone at 24 h after surgery. PCA, patient-controlled analgesia.

Two studies (17,20) (n=166) provided specific data for analysis of PGESM at 24 h after surgery. We selected the fixed-effect model to perform the meta-analysis since there were no significant heterogeneities (effective, χ^2^=0.46, P=0.50, I2= 0%; ineffective, χ^2^= 0.15, P= 0.70, I^2^= 0%). The incidence of ‘effective’ and ‘ineffective’ results in the analgesic effect evaluation was significantly different between the two groups [RR=1.41, 95% CI (1.13–1.75), P=0.002; and RR=0.43, 95% CI (0.26–0.72), P=001, respectively; Fig. 2].

#### Patient global evaluation 48 h after the initial dose of parecoxib

**Figure 3.**
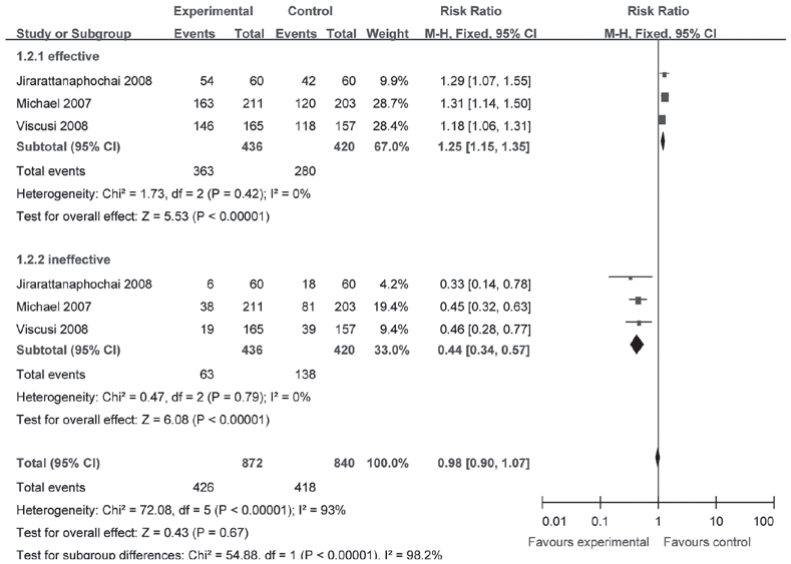
Meta-analysis of patients’ global evaluation of study medication of PCA combined with parecoxib sodium and PCA alone at 48 h after surgery. PCA, patient-controlled analgesia.

Three studies (15,16,19) (n=868) provided specific data for analysis of PGESM at 48 h after surgery. We selected the fixed-effect model to perform the meta-analysis since there were no significant heterogeneities (effective, χ^2^=1.73, P=0.42, I2= 0%; ineffective χ^2^= 0.47, P= 0.79, I^2^= 0%). The incidence of ‘effective’ and ‘ineffective’ results in the analgesic effect evaluation was significantly different between the two groups [RR=1.25, 95% CI (1.15–1.35), P<0.00001; and RR=0.44, 95% CI (0.34–0.57), P<0.00001, respectively; Fig. 3].

#### Patient global evaluation 72 h after the initial dose of parecoxib

**Figure 4.**
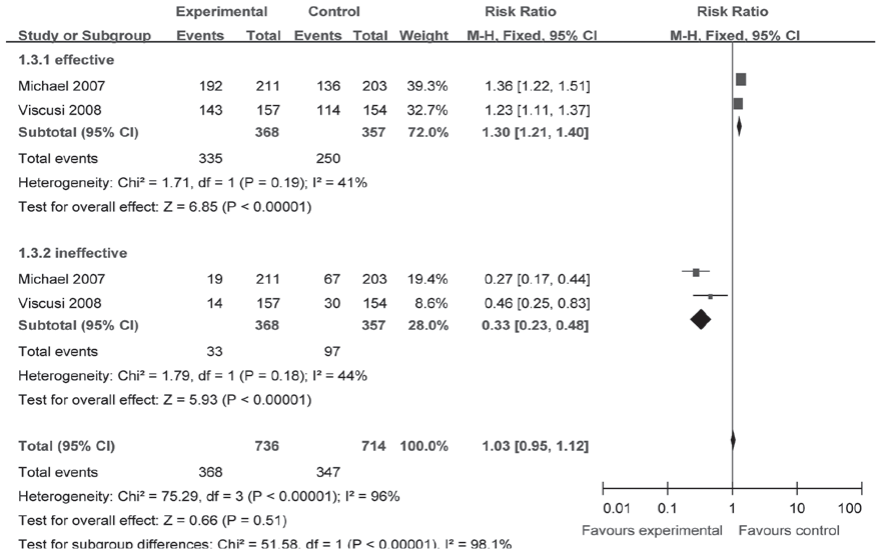
Meta-analysis of patients’ global evaluation of study medication of PCA combined with parecoxib sodium and PCA alone at 72 h after surgery. PCA, patient-controlled analgesia.

Two studies (15,16) (n=748) provided specific data for analysis of PGESM at 72 h after surgery. We selected the fixed-effect model to perform the meta-analysis since there were no significant heterogeneities (effective, χ^2^=1.71, P= 0.19, I^2^= 41.4%; ineffective χ^2^=1.79, P=0.18, I ^2^= 44.2%). The incidence of ‘effective’ and ‘ineffective’ results in the analgesic effect evaluation was significantly different between the two groups [RR=1.30, 95% CI (1.21–1.40), P<0.00001; and RR=0.33, 95% CI (0.23–0.48), P<0.00001, respectively; Fig. 4].

### Comparisons of safety

**Figure 5.**
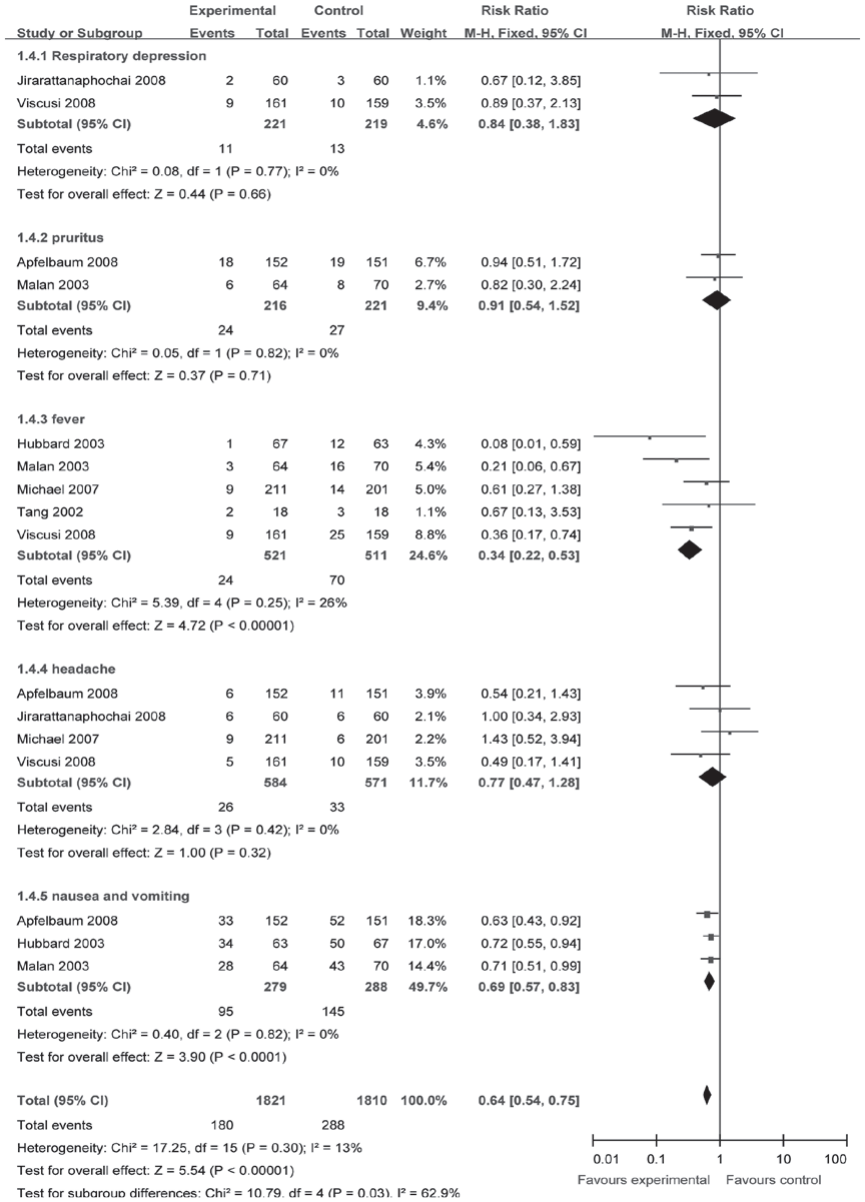
Meta-analysis of adverse drug reactions after surgery of PCA combined with parecoxib sodium and PCA alone. PCA, patient-controlled analgesia.

We selected the fixed-effect model to perform the meta-analysis since there were no significant heterogeneities (respiratory depression, χ^2^= 0.08, P= 0.77, I2= 0%; pruritus, χ^2^= 0.05, P= 0.82, I^2^= 0%; fever, χ^2^=5.39, P= 0.25, I^2^=25.8%; headache, χ^2^=2.84, P=0.42, I^2^=0%; nausea and vomiting, χ^2^= 0.40, P= 0.82, I^2^=0%). Two (15,18) (n=454), two (14,18) (n=437) and four studies (14–16,19) (n=1171) provided data of respiratory depression, pruritus and headache, respectively. The incidence of respiratory depression, pruritus and headache between the treatment and control groups was not significantly different [RR= 0.84, 95% CI (0.38–1.83), P=0.66; RR=0.91, 95% CI (0.54–1.52), P=0.71; and RR=0.77, 95% CI (0.47–1.28), P=0.32, respectively; Fig. 5].

Five (15–18,20) (n=1048) and three studies (14,18,20) (n=567) provided data on fever, and nausea and vomiting, respectively. The incidences of fever, and nausea and vomiting between the treatment and control groups were significantly different [RR= 0.34, 95% CI (0.22–0.53), P<0.00001; and RR=0.69, 95% CI (0.57–0.83), P<0.00001, respectively; Fig. 5].

## Discussion

NSAIDs are known to induce analgesia mainly via inhibition of COX. Parecoxib exhibits anti-inflammatory, analgesic and antipyretic properties in animal models and humans due to inhibition of prostanoid synthesis primarily by affecting COX-2. Although the inhibition of COX in the periphery is commonly accepted as the primary mechanism, experimental and clinical data suggest a potential role for central COX inhibition to produce antinociception and reduce hypersensitivity. Additionally, it has double analgesic actions (21). Multimodal analgesia, where opioids, including morphine, are administered with a non-opioid, is often used to reduce opioid-related adverse effects, including postoperative nausea and vomiting, drowsiness, respiratory depression and gastrointestinal and bladder dysfunction (22). The underlying principle is that the different modes of action of morphine and the non-opioid drug allow optimum analgesia to be maintained with a lower dose of morphine and consequently a lower incidence of morphine-related adverse effects (23).

We conducted the current meta-analysis to compare the efficacy and safety of parecoxib sodium plus PCA with PCA alone for acute postoperative pain. The results of the meta-analysis indicated that the efficacy of PCA combined with parecoxib sodium (successively injected for <3 days intravenously) was superior to that of PCA alone with a statistically significant difference. After 24, 48 and 72 h of the initial dose of 40 mg parecoxib i.v., the percentage of ‘effective’ treatment as described by PGESM was higher compared with that of the control group; the percentage of ‘ineffective’ treatment was lower compared with that of the control group. Moreover, PCA plus parecoxib sodium reduced the incidence of postoperative fever, nausea and vomiting; however, it did not significantly reduce the incidence of respiratory depression, pruritus and headache. The incidence of postoperative bleeding, urinary retention, digestive tract ulcer, pulmonary embolism, massive hemorrhage and cardiovascular events in all the included studies were extremely low, which demonstrated an improved security of parecoxib sodium. However, a previous study indicated that there was a reduction in 24-h morphine consumption, leading to a reduction in morphine-related adverse effects when COX-2 inhibitors were administered in addition to PCA morphine following surgery, with no clear difference between them (23). Therefore, our findings indicate that parecoxib sodium may be beneficial in pain relief following surgery; however, further studies are required to confirm this.

Certain limitations affecting the results of the current meta-analysis should be taken into account. Firstly, our findings may be affected by the quality of trials included in the meta-analysis. A well-designed randomized controlled trial requires a thorough understanding of randomization so that better results are achieved. However, none of the included trials clarified allocation concealment clearly. All studies were only assessed to be a Jadad score B in terms of methodology. Secondly, this meta-analysis is based on a relatively small number of RCTs and we acknowledge that using a limited number of studies raises the possibility of a second-order sampling error (24). Thirdly, the distinct differences in administration times, dose, treatment course, different surgery and initial pain level of patients used exist (Table I), which may affect the consistency of effects across those included studies.

In conclusion, although certain limitations exist in this meta-analysis, based on the results of our meta-analysis, we identified that parecoxib is an effective and relatively safe option for acute postoperative pain. However, further high quality RCTs are required to determine the long-term effects of parecoxib for postoperative pain.

## Figures and Tables

**Table I.** Study characteristics and quality assessment of included RCTs.

Study (ref.)	Sample size (n)		Intervention measures		Methodological quality evaluation			
	T	C	T	C	Randomization	Blinding	Allocation concealed	Quality grade
Apfelbaum 2008 (13)	T_1_:151	151	T_1_: 40+20 mg qd i.v. prior before the end of surgery	NS	Computer stochastic	Double blinding	Unclear	B
	T_2_:152		T_2_: 40+20 mg bid i.v. before the end of surgery					
Michael 2007 (14)	211	203	40+20 mg bid i.v. before the end of surgery	NS	Computer stochastic	Double blinding	Unclear	B
Viscusi 2008 (15)	T_1_:166	167	T_1_:40+20 mg qd i.v. before the end of surgery	NS	Computer stochastic	Double blinding	Unclear	B
	T_2_:167		T_2_: 40+20 mg bid i.v. before the end of surgery					
Tang 2002 (16)	T_1_:19	18	T_1_: 20 mg bid i.v. before the end of surgery	NS	Computer stochastic	Double blinding	Unclear	B
	T_2_:18		T_2_: 40 mg bid i.v. before the end of surgery					
Malan 2003 (17)	T_1_:67	70	T_1_: 20 mg bid i.v. before the end of surgery	NS	Computer stochastic	Double blinding	Unclear	B
	T_2_:64		T_2_: 40 mg bid i.v. before the end of surgery					
Jirarattanaphochai 2008 (18)	60	60	40+40 mg bid i.v. before the end of surgery	NS	Computer stochastic	Double blinding	Unclear	B
Hubbard 2003 (19)	T_1_:65	63	T1: 20 mg bid i.v. before the end of surgery	NS	Computer stochastic	Double blinding	Unclear	B
	T_2_:67		T_2_: 40 mg bid i.v. before the end of surgery					

RCTs, randomized controlled trials; T, treatment group; C, control group; NS, not specified.
